# Chronic urticaria with angioedema

**DOI:** 10.11604/pamj.2021.39.253.30271

**Published:** 2021-08-19

**Authors:** Sarah Nasser, Abraham Husseini

**Affiliations:** 1 Wayne State University School of Medicine, Department of Internal Medicine and Pediatrics, Detroit, Michigan, United States of America,; 2 Detroit Medical Center, Department of Internal Medicine and Pediatrics, Detroit, Michigan, United States of America

**Keywords:** Urticaria, angioedema, chronic

## Image in medicine

A 25-year-old gentleman with no past medical history presents with a 1-2 year complaint of intermittent episodes of a diffuse pruritic raised skin rash on his limbs and trunk (A). He reports this has happened to him 5-6 times in total and is typically associated with him awakening to find the room too warm. He brings up the issue for the first time because he also had angioedema of the lips that persisted for approximately 24 hours (B). There were no other identifiable food or environmental triggers, and he denied any headaches, dyspnea, nausea, vomiting. The patient was reassured that this is chronic urticaria, prescribed cetirizine as needed, and informed that this will likely resolve on its own in a few years.

**Figure 1:**
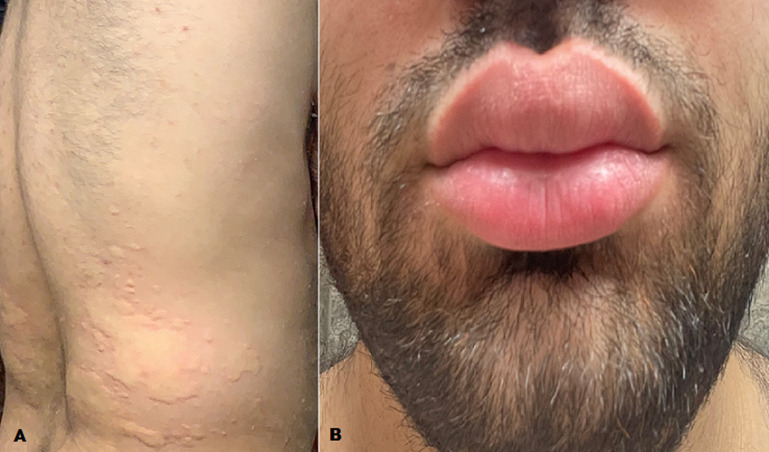
A) urticaria on trunk; B) angioedema

